# EMERGENCY DEPARTMENT AND HOSPITAL USE AMONG COGNITIVELY VULNERABLE MEDICARE ADVANTAGE ENROLLEES

**DOI:** 10.1093/geroni/igae098.1042

**Published:** 2024-12-31

**Authors:** Richard Fortinsky, Xiaomin Lu, James Grady, Julie Robison, David Steffens, George Kuchel

**Affiliations:** University of Connecticut, Farmington, Connecticut, United States; University of Connecticut, Storrs, Connecticut, United States; University of Connecticut, Farmington, Connecticut, United States; University of Connecticut, Farmington, Connecticut, United States; University of Connecticut, Farmington, Connecticut, United States; University of Connecticut, Farmington, Connecticut, United States

## Abstract

Medicare Advantage (MA) health insurance plans now cover more individuals than original Medicare. Very little is known about patterns of emergency department (ED) visits and hospitalizations among MA enrollees, especially those living with cognitive vulnerability due to dementia, depression, and/or recent delirium. Therefore, we explored factors associated with ED visits and hospitalizations among older adults enrolled in an MA plan and living at home with cognitive vulnerability. Our study cohort includes 406 adults age >65 who completed a 12-month clinical trial; baseline data collected by interview were linked with ED and hospital use data from medical claims for 12 months following study baseline. Predictors, all from study baseline, included age group, sex, race, ethnicity, educational level, financial strain level, number of medical comorbidities, and cognitive vulnerability type: dementia only; depression only; dementia and depression; or delirium with dementia and/or depression. Cohort characteristics: 58.4% female; mean (SD) age=76.1 (7.0); 92.1% White individuals; 4.7% Black individuals; 39.4% had >1 ED visit and 26.4% had >1 hospitalization. Multivariate logistic regression results revealed that the likelihood of experiencing an ED visit was positively associated with number of medical comorbidities at baseline (AOR=1.18, p=0.001), and participants with depression only were less likely than those with recent delirium at baseline to experience an ED visit (AOR=0.33, p=0.02). These same predictors were associated with hospitalization likelihood (AOR=1.14, p=0.01; and AOR=0.27, p=0.01, respectively). We conclude that medical comorbidity burden as well as delirium history are important risk factors for subsequent ED visits and hospitalizations in this MA cohort.

